# Surgical resection of cardiac myxoma—a 30-year single institutional experience

**DOI:** 10.1186/s13019-017-0583-7

**Published:** 2017-03-27

**Authors:** Kyo Seon Lee, Gwan Sic Kim, Yochun Jung, In Seok Jeong, Kook Joo Na, Bong Suk Oh, Byung Hee Ahn, Sang Gi Oh

**Affiliations:** Department of Thoracic and Cardiovascular Surgery, Chonnam National University Hospital, Chonnam National University School of Medicine, 42, Jebong-ro, Dong-gu, Gwangju, 15772 South Korea

**Keywords:** Myxoma, Benign cardiac tumor

## Abstract

**Background:**

Primary cardiac tumors are rare and myxoma constitutes the majority. The present study summarizes our 30-year clinical outcomes of surgical myxoma resection.

**Methods:**

Between January 1986 and December 2015, 93 patients (30 men, 63 women; mean age, 54.7 ± 16.6 years) underwent surgical myxoma resection. The most common origin site was the left atrium. Surgery was performed via a biatrial approach in 74.2%, atrial septotomy through right atriotomy in 17.2%, and left atriotomy only in 8.6%. Mean myxoma size based on longest length was 4.73 ± 1.92 cm (range, 1.2–11.0 cm).

**Results:**

The mean follow-up duration was 9.9 ± 7.8 years (range, 0–29 years). In-hospital mortality was 3.2%. The most common postoperative complication was atrial fibrillation (4.3%). The 5-, 10-, and 30-year survival rates were 92.9%, 87.2%, and 75.5%, respectively. Recurrence occurred in two patients (2.1%), which were detected at 20 and 79 months after the first surgery, respectively.

**Conclusions:**

Long-term survival after myxoma resection was excellent and recurrence was rare. Based on our experience, surgical method did not affect the outcome.

## Background

Primary cardiac tumors are rare and approximately 70% are benign. Myxoma is the only relatively common primary heart neoplasm [1, 2], which is often found on 2-dimensional (2D) echocardiography. Myxoma occurs in all ages and is 2 to 3 times more common in women than in men. It can cause mild constitutional symptoms, such as fever, weight loss, myalgia, or arthralgia, to serious hemodynamic derangement, depending on its location and size, which can lead to disastrous embolic symptoms. Therefore, if possible, prompt surgical resection is recommended.

The objective of this study was to assess our long-term clinical outcomes of intracardiac myxoma resection in a consecutive series of 93 patients.

## Methods

From January 1986 to December 2015, 93 patients with cardiac myxoma underwent surgery at our institution. Myxoma was 2 times more common in women (63 patients, 67.7%) than in men (30 patients, 32.3%), and the mean age of the patients was 54.7 ± 16.6 years (range, 1–78 years).

The origin site of the tumor was the left atrium (LA) in 86 patients (92.5%), right atrium in 4 (4.3%), and left ventricle in 2 (2.2%). Multiple myxomas in both atria and the right ventricle were present in one patient (1.1%); his family history of myxoma was not identified. Among the tumors originating in the LA, the most common implant site was the fossa ovalis (76 patients, 81.7%). A pedunculated mass was found in 63 patients (67.6%), while the other 30 patients (32.3%) had a sessile mass. Other cardiac surgeries were performed in 19 patients (20.4%), with mitral valve surgery being the most common procedure (Table 1). Four patients underwent mitral valve surgery due to the organic change of the mitral valve (rheumatic mitral stenosis in 1 and degenerative mitral insufficiency in 3). Four patients had myxoma-related mitral valve lesions. Four patients had mitral annular enlargement and one of them had a leaflet fibrotic change.

**Table 1:** Cardiac surgeries performed concurrently with myxoma resection

Surgery	n (%)
Maze procedure	4 (4.3)
MVR	3 (3.2)
MVP	2 (2.2)
CABG	2 (2.2)
MVP + TVP	1 (1.1)
MVP + Maze procedure	1 (1.1)
MVR + Maze procedure	1 (1.1)
AVR + Maze procedure	1 (1.1)
PFO closure	1 (1.1)
RVOT release	1 (1.1)
VSD closure	1 (1.1)
Lung wedge resection	1 (1.1)

*Abbreviations*: *AVR* aortic valve replacement, *CABG* coronary artery bypass grafting, *MVP* mitral valvuloplasty, *MVR* mitral valve replacement, *PFO* patent foramen ovale, *RVOT* right ventricular outflow tract, *TVP* tricuspid valvuloplasty, *VSD* ventricular septal defect

The most common symptom was dyspnea in 36 patients, 19 of whom had severe dyspnea greater than New York Heart Association functional classification III. Other cardiac or embolic symptoms included chest pain, palpitation, syncope, and stroke. Constitutional symptoms, such as fever, cough, weight loss, and headache, occurred in five patients, whereas 18 patients had an intracardiac mass detected incidentally without any symptoms (Table 2).

**Table 2:** Preoperative clinical symptoms and signs

Symptom/sign	n (%)
Dyspnea	36 (38.7)
Chest pain	20 (21.5)
Palpitation	8 (8.6)
Stroke	10 (10.8)
Syncope	5 (5.4)
Fever	1 (1.1)
Cough	1 (1.1)
Headache	1 (1.1)
Weight loss	1 (1.1)
Nausea	1 (1.1)
Asymptomatic	18 (19.4)

All myxomas were diagnosed by transthoracic echocardiography. However, if tumors other than myxomas were suspected, we performed transesophageal echocardiography, chest computed tomography, or magnetic resonance imaging.

Sixty-five patients underwent elective surgery, whereas 28 patients with severe symptoms or embolic risk underwent emergency surgery. Regarding elective surgery, preoperative coronary angiography was performed routinely in all male patients over 40 years of age and postmenopausal women. All surgeries were performed through median sternotomy. Routinely, the ascending aorta and both caval veins were cannulated, and a cardioplegic solution was infused in an antegrade or retrogade fashion. Retrograde cardioplegia infusion was used when aortic insufficiency was found on preoperative 2D echocardiography, or the operation time was expected to take longer due to combined operation like valve surgery. Surgery was performed via a biatrial approach in 69 patients (74.2%), transseptal approach with right atriotomy and septotomy in 16 (17.2%), and left atriotomy only in eight patients (8.6%), depending on the size and location of the mass confirmed on preoperative 2D echocardiography.

Simple myxoma resection including the endocardium and attached stalk without any need to repair was performed in 17 patients, direct closure of the defect area was performed in 47, and patch closure with autopericardium or prosthetic material was performed in 29. If the expected defect was too large to close directly, based on preoperative 2D echocardiography, autopericardium was harvested before pericardiotomy and fixed with glutaraldehyde for 5 min. If the defect was recognized as too large to close directly, prosthetic material was used for closure; bovine pericardium (Supple Peri-Guard®; Synovis Surgical Innovations, St. Paul, MN) or a dacron patch (Bard® Sauvage® Filamentous Knitted Polyester Fabric; Bard Peripheral Vascular Inc., AZ) was used according to the preference of the surgeon. Mean cardiopulmonary bypass time was 80.7 ± 39.0 min (range, 19–231 min), and mean aortic cross-clamping time was 51.3 ± 27.5 min (range, 9–153 min).

**Fig. 1:**
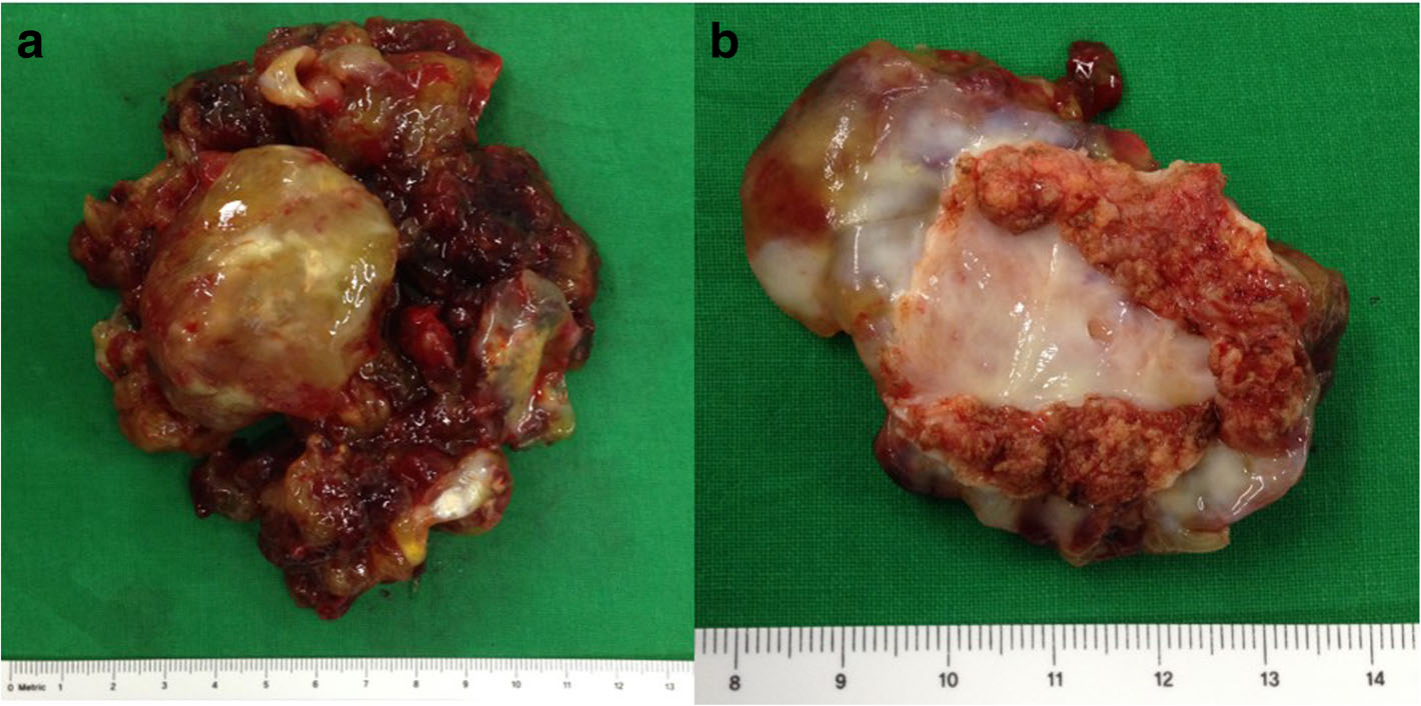
Images of a giant myxoma. **a** The resected myxoma is huge. **b** The resected myxoma has a broad base of approximately 4 cm in diameter

**Fig. 2:**
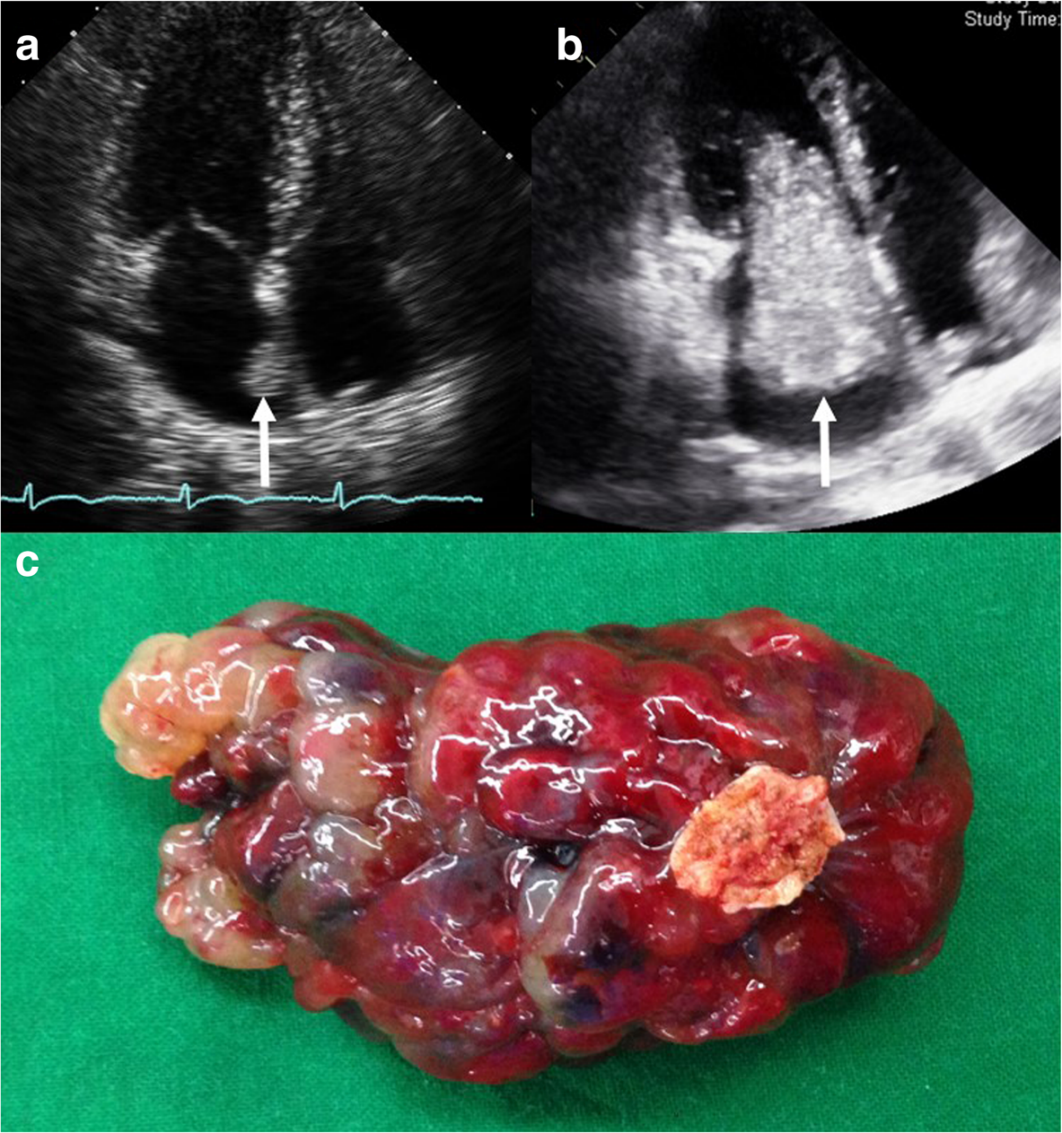
Images of a rapidly growing myxoma. **a** A small myxoma is attached to the left atrial side of the fossa ovalis. **b** An enlarged myxoma passes in and out of the mitral valve according to the cardiac cycle. **c** The resected myxoma is sessile and has a narrow base

Mean myxoma size based on the longest length was 4.73 ± 1.92 cm (range, 1.2–11.0 cm), and a giant myxoma over 10 cm in length was found in two patients (10.0 × 4.0 × 2.0 cm and 11.0 × 10.0 × 3.5 cm) (Fig. 1). One patient was recommended surgical resection of a mass located on the fossa ovalis measuring approximately 1.66 × 1.6 × 1.0 cm. However, she refused the surgery and revisited 15 months later with severe dyspnea resulting from functional mitral stenosis. Preoperative 2D echocardiography revealed that the mass had grown to 7.7 × 3.38 × 1.0 cm (Fig. 2). Surgical resection was performed successfully.

Long-term survival was assessed based on the National Health Insurance database. Results are presented as mean ± SD. Long-term cumulative survival was analyzed using the Kaplan-Meier method. Analyses were performed using PASW version 18.0 (IBM Corporation, Armonk, NY, USA). This study was approved by the Institutional Review Board of Chonnam National University Hospital (IRB No. CNUH-2016-217), which waived the requirement for informed patient consent because of the retrospective nature of the study.

## Results

The most common postoperative complications were atrial fibrillation in four patients and wound infection in 3. Newly developed atrial fibrillation occurred after a biatrial or transseptal approach in two patients each (*p* = 0.136), with no conduction disturbance postoperatively. Other complications included low cardiac output syndrome, complete atrioventricular block, cerebral hemorrhage, hoarseness, pneumonia, and acute cholangitis. In-hospital mortality occurred in three patients (3.2%) due to postoperative pneumonia, low cardiac output syndrome (the patient had a ventricular septal defect with a myxoma in the left ventricle), or metabolic acidosis and dilutional coagulopathy after massive transfusion caused by intraoperative coronary sinus rupture.

**Fig. 3:**
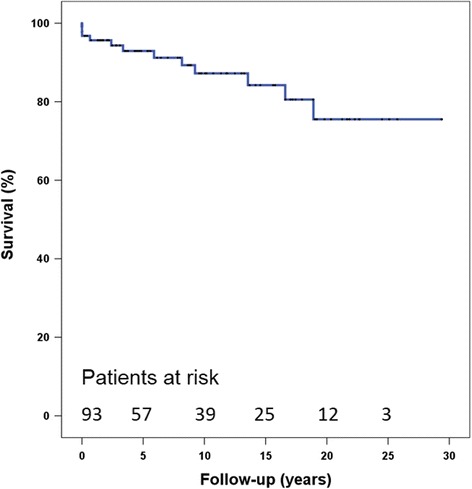
Long-term cumulative survival after myxoma resection

Mean follow-up duration was 9.9 ± 7.8 years (range, 0–29 years), and 12 of 93 patients became ineligible for the National Health Insurance; these 12 patients were regarded as dead because change in nationality was not identified during this period. The 5-, 10-, and 30-year survival rates were 92.9, 87.2 and 75.5%, respectively (Fig. 3). Myxoma recurred in two patients (2.1%) during the follow-up period. One patient with multiple myxomas in the LA, right atrium, and right ventricle underwent a reoperation 79 months after the primary operation. The recurred masses were located on the LA roof, right atrial septum, and trabecula of the right ventricle. All masses were widely resected, the septal defect was reconstructed using a Dacron patch, and plication was performed for LA roof repair. The other patient experienced cerebral infarction 20 months after the primary surgery, and 2D echocardiography revealed a mass on the previously resected margin; myxoma could not be ruled out completely. The patient refused a reoperation, and the mass did not grow during the follow-up period. These recurred patients underwent direct closure of the defect after tumor removal. No recurred tumor was evident in patients with simple myxoma resection or patch closure of the defect. However, this was not statistically significant because there were few recurred cases.

## Discussion

Myxoma is the most common primary cardiac tumor, which frequently occurs in middle age and is more common in women than in men [2–5]. In our data, the mean age of the patients was 54.7 years at the time of surgery, and 86% were aged over 40 years. In addition, myxoma was 2 times more common in women than in men.

Myxomas vary in size, but the growth rate is difficult to document because they are usually removed after diagnosis [6–9]. However, in our study, the one patient who underwent surgery 15 months after initial diagnosis showed a rapid growth rate of 0.41 cm/month.

Although operative mortality is reported to be within 5%, the rate is increased with myxoma occurring in the ventricle [3, 5, 10, 11]. In our data, in-hospital mortality occurred in three patients. One patient died from low cardiac output syndrome after extensive resection of the left ventricle, while the other two patients died from pneumonia or coronary sinus rupture, which may occur after cardiac surgery. Pneumonia after cardiac surgery is one of the most common causes of postoperative mortality, and coronary sinus rupture during retrograde cardioplegia infusion is also a possible complication during cardiac surgery. These complications may occur due to the nature of cardiac surgery rather than myxoma itself. Although myxoma resection is relatively simple compared with other cardiac surgeries, it does require cardiopulmonary bypass during cardiac arrest. Therefore, postoperative complications may occur as in other more complex cardiac surgeries, and should be recognized.

Regarding the surgical approach, a biatrial approach is helpful to determine the correct resection margin by confirming the tumor pedicle under direct visualization, to minimize manipulation of the tumor, to find hidden myxomas by inspection of all heart chambers, and to secure the septal defect after resection of a tumor located on the atrial septum [12]. On the other hand, a transseptal approach through right atriotomy may provide adequate mass exposure and, as a result, low recurrence and easy repair of a single incision on the right atrium [13]. However, if the myxoma is sessile, has a broad base, and is attached to the atrial septum, a biatrial approach, rather than a transseptal approach, may reduce the risk of injury and tumor emboli. Even though a transseptal approach is simpler than a biatrial approach, it is only useful when the myxoma is pedunculated and has a narrow stalk. We primarily used biatrial incisions, but, a transseptal approach or left atriotomy only was used for resection of small myxomas confirmed on preoperative 2D echocardiography. Our institution does not have a guideline regarding the incision site as it relates to tumor size. However, if the largest tumor is less than 2 ~ 3 cm in size, wherein the tumor fully can be removed through a transseptal incision without breaking, the base of the stalk is narrow, and the motion of the tumor is active according to the heart beat on 2D echocardiography, we use a transseptal approach. In all other cases, a left atriotomy should be performed first. The position and shape of the tumor is evaluated, and, if the tumor can be removed without manipulation, it is removed without an additional incision. If the stalk is short or of a sessile form, we perform an additional right atriotomy to secure the resection margin. Because all tumors can be detected on preoperative transthoracic echocardiography due to advances in imaging techniques, and additional intracardiac evaluation is performed during surgery using transesophageal echocardiography, especially when left atriotomy only is performed, additional cardiac incisions for evaluation of all cardiac chambers may not be necessary.

The recurrence rate after myxoma resection has been reported to be less than 5% [3–5, 12]. In our data, two patients (2.1%) had recurrence. One patient had multiple myxomas and underwent a reoperation. The other patient was suspected to have recurrence on the atrial septum, but it was not confirmed histologically. Because we could not rule out myxoma completely, we regarded it as recurrence on the previously resected margin. Myxoma recurrence is caused by incomplete resection or tumor seeding during mass manipulation [14]. Our two cases of recurrence were thought to be caused by incomplete primary resection because they were located on the resection margin. Therefore, some authors insist on wide resection to prevent recurrence following incomplete resection [15]. However, other reports claim that simple tumor resection is sufficient because of the low recurrence rate [4, 16]. Our recurred patients underwent full-layer resection including the stalk and primary closure, and no patient who underwent only simple myxoma resection without defect repair was recurred. Therefore, it is thought that all myxoma patients are not necessary taken full-layer resection. However, in cases of myxoma occurring at a relatively young age, with ventricular origin, family history, Carney complex, or multiple myxomas, full-layer wide resection is recommended because of the increased recurrence rate [3, 5, 12, 17].

Our study has several limitations. First, this was a retrospective study. However, this study design was inevitable because cardiac tumors are extremely rare and early surgical resection is the treatment of choice. Second, we estimated mortality as total death without considering cardiac or sudden death separately. Although this does not reflect disease-related mortality specifically, it is thought that total death lowers selection bias.

## Conclusions

Clinical outcomes of myxoma resection are acceptable, but complications associated with cardiopulmonary bypass or cardiac arrest should be recognized. Although recurrence is low and is not affected by surgical method, wide resection is generally recommended in patients with increased likelihood of recurrence.

## Abbreviations

2D: 2 dimentional
LA: Left atrium
